# Dynamic protein deacetylation is a limited carbon source for acetyl-CoA–dependent metabolism

**DOI:** 10.1016/j.jbc.2023.104772

**Published:** 2023-05-02

**Authors:** Ioana Soaita, Emily Megill, Daniel Kantner, Adam Chatoff, Yuen Jian Cheong, Philippa Clarke, Zoltan Arany, Nathaniel W. Snyder, Kathryn E. Wellen, Sophie Trefely

**Affiliations:** 1 Cardiovascular Institute, Perelman School of Medicine, University of Pennsylvania, Philadelphia, Pennsylvania, USA; 2 Center for Metabolic Disease Research, Lewis Katz School of Medicine, TempleUniversity, Philadelphia, Pennsylvania, USA; 3 Epigenetics and Signalling Programs, Babraham Institute, Cambridge, UK; 4 Department of Cancer Biology, Perelman School of Medicine, University of Pennsylvania, Philadelphia, Pennsylvania, USA; 5 Abramson Family Cancer Research Institute, Perelman School of Medicine, University of Pennsylvania, Philadelphia, Pennsylvania, USA

**Keywords:** acetylation, acetyl-coenzyme A, histone, stable isotope tracing, acetate, metabolism

## Abstract

The ability of cells to store and rapidly mobilize energy reserves in response to nutrient availability is essential for survival. Breakdown of carbon stores produces acetyl-CoA (AcCoA), which fuels essential metabolic pathways and is also the acyl donor for protein lysine acetylation. Histones are abundant and highly acetylated proteins, accounting for 40% to 75% of cellular protein acetylation. Notably, histone acetylation is sensitive to AcCoA availability, and nutrient replete conditions induce a substantial accumulation of acetylation on histones. Deacetylation releases acetate, which can be recycled to AcCoA, suggesting that deacetylation could be mobilized as an AcCoA source to feed downstream metabolic processes under nutrient depletion. While the notion of histones as a metabolic reservoir has been frequently proposed, experimental evidence has been lacking. Therefore, to test this concept directly, we used acetate-dependent, ATP citrate lyase–deficient mouse embryonic fibroblasts (*Acly*^*−/−*^ MEFs), and designed a pulse-chase experimental system to trace deacetylation-derived acetate and its incorporation into AcCoA. We found that dynamic protein deacetylation in *Acly*^*−/−*^ MEFs contributed carbons to AcCoA and proximal downstream metabolites. However, deacetylation had no significant effect on acyl-CoA pool sizes, and even at maximal acetylation, deacetylation transiently supplied less than 10% of cellular AcCoA. Together, our data reveal that although histone acetylation is dynamic and nutrient-sensitive, its potential for maintaining cellular AcCoA-dependent metabolic pathways is limited compared to cellular demand.

Cells stockpile energy in intracellular stores for survival across variations in nutrient supply. Under nutrient deprivation, mobilization of intracellular energy stores generates acetyl-CoA (AcCoA), an essential metabolite in pathways required for survival. AcCoA is also the acyl donor for protein acetylation, thus directly influencing protein signaling and epigenetic regulation (1, 2). Histones are highly abundant proteins, and histone lysine acetylation occupancy has been measured at 10-fold that of cytosolic and mitochondrial proteins (3). Quantitative studies of protein acetylation have attributed between 40% and 75% of cellular acetylation to histones/nuclear proteins (4, 5, 6) (Table S2). Notably, bulk histone acetylation (Hac) is sensitive to AcCoA, and the supply of AcCoA for acetylation of specific Hac marks is important for gene regulation in contexts such as cell differentiation (7, 8, 9, 10, 11, 12, 13, 14). However, whether Hac plays roles in cell physiology beyond gene regulation and DNA repair remains poorly understood (15, 16, 17, 18, 19, 20).

Homeostatic turnover of Hac occurs over a timeframe of minutes to hours in mammalian cell culture under stable nutrient conditions. This dynamic turnover is facilitated by the removal and conversion of lysine acetylation to free acetate through the action of lysine deacetylases (KDACs) (21, 22, 23). Free acetate can then be converted to AcCoA by cytosolic/nuclear acyl-CoA synthetase short chain family member 2 (ACSS2) and recycled for the acetylation of specific histone marks (17, 24, 25, 26, 27). The contribution of histone deacetylation to whole-cell AcCoA and AcCoA-dependent metabolism, however, is unknown. The potential for bulk acetyl sequestration under nutrient-replete conditions has led to speculation that Hac may serve as an acetate reservoir that can be mobilized as a metabolic source beyond the nucleus (15, 16, 18, 19).

To test the capacity for histone deacetylation to directly contribute to cellular AcCoA, we designed an experimental system to trace deacetylation-derived acetate incorporation into AcCoA. We employed ^13^C_2_-acetate in mouse embryonic fibroblasts deficient in ATP citrate lyase (*Acly*^*−/−*^ MEFs), which are primarily dependent on acetate for AcCoA and acetylation (27). We measured the maximal contribution of protein-bound acetate carbon units to AcCoA by inducing deacetylation after maximizing acetylation. We found that deacetylation transiently supplied approximately 10% (9.1%) of cellular AcCoA. Our results demonstrate that protein deacetylation (encompassing histone and, to a lesser extent, nonhistone deacetylation) can directly contribute to AcCoA but is not acutely a major source of total cellular AcCoA.

## Results

### Estimating the acetate storage capacity of histones

**Figure 1:**
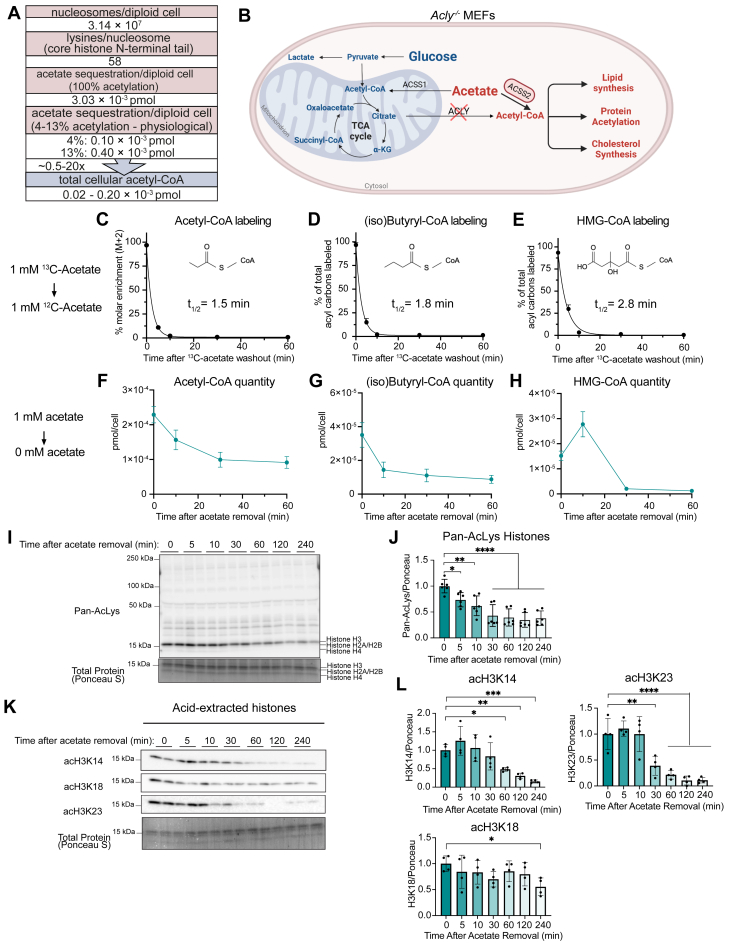
**Acyl-CoA pools and Hac respond rapidly to acetate in *Acly***^***-/-***^**MEFs.***A*, estimates of total and physiological acetate sequestration per human diploid cell compared to AcCoA pool size. *B*, compartmentalized glucose and acetate metabolism in *Acly*^*−/−*^ MEFs. *C*–*E*, loss of ^13^C enrichment after replacement of 1 mM ^13^C_2_-acetate with unlabeled acetate for indicated acyl-CoAs. N = 9 across three independent experiments. *F*–*H*, quantitation of indicated acyl-CoAs following acetate removal. N = 9 across three independent experiments. *I*, representative IB of pan-Kac in cell extracts after 1 mM acetate removal. *J*, quantitation of pan-Kac signal representing Hac (H3 = 15 kDa; H2A/H2B = 14 kDa; and H4 = 11 kDa), normalized to total protein signal. N = 6 across three independent experiments. *K*, representative IB of indicated histone lysine sites in acid-extracted histones after 1 mM acetate removal. *L*, quantitation of Kac in (**K)** normalized to total protein. N = 4 across two independent experiments. All data indicated as mean ± SD. Statistical analysis in *J* and *L* was by one-way ANOVA followed by Dunnett’s Multiple Comparison test. ns = not significant. *p* value < 0.05 (∗), *p*-value < 0.01 (∗∗), *p*-value < 0.001 (∗∗∗), and *p* value < 0.0001 (∗∗∗∗). *B*, created with BioRender.com. AcCoA, acetyl-CoA; ACLY, ATP citrate lyase; Hac, histone acetylation; IB, immunoblot; Kac, acetyl lysine; MEF, mouse embryonic fibroblasts.

**Figure 2:**
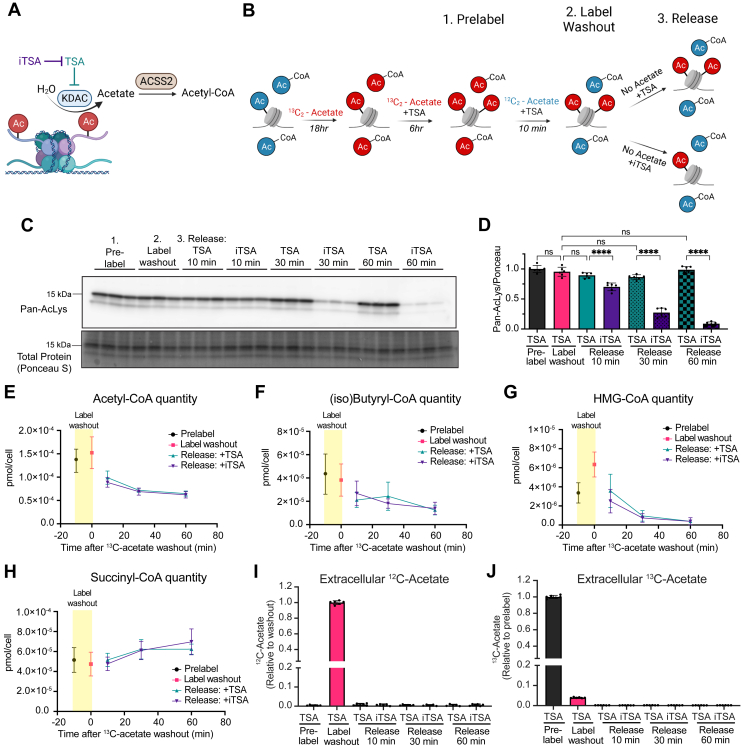
**Deacetylation does not substantially impact cellular acyl-CoA levels.***A*, schematic of histone deacetylation contributing to AcCoA. *B*, schematic of Hac labeling in pulse-chase experiment. *C*, representative pan-Kac IB of whole-cell extracts during pulse-chase experiment. *D*, quantitation of pan-Kac signal normalized to total protein signal. N = 6 across two independent experiments. *E*–*H*, quantitation of indicated acyl-CoAs in pulse-chase experiment. N = 7 across two independent experiments. *I* and *J*, measurement of extracellular (*I*) ^12^C-acetate and (*J*) ^13^C-acetate in pulse-chase experiment. N = 6 across two independent experiments. All data indicated as mean ± SD. Statistical analysis in (*D*) was by one-way ANOVA followed by Tukey’s Multiple Comparison test with relevant comparisons indicated. ns = not significant. *p*-value < 0.0001 (∗∗∗∗). *A* and *B* created with BioRender.com. AcCoA, acetyl-CoA; Hac, histone acetylation; IB, immunoblot; Kac, acetyl lysine.

We first sought to estimate the acetate storage capacity of histones under physiological conditions. We focused on histone N-terminal lysine sites, since Hac occurs primarily on lysine residues of disordered N-terminal tails (28, 29). We multiplied the number of N-terminal lysine residues (H2A, H2B, H3, and H4) by estimates for the total number of each histone per human diploid cell, resulting in 3.03 × 10^−3^ pmol acetate per cell (Fig. 1*A*; see Table S1 for calculations) (30, 31, 32). We calculated that a similar amount of acetate can be stored on histones in a mouse diploid cell (Table S1) (33). Total cellular AcCoA has been measured in the range of 2 × 10^−5^ to 2 × 10^−4^ pmol/cell (Figs. 1 and 2) (8, 34). Therefore, at maximum acetylation capacity, we estimate that Hac has the potential to provide 10 to 100 times cellular AcCoA quantity, agreeing with previous estimates (16, 19). However, measurements of acetylation occupancy at specific sites indicate that physiological occupancy averages between 4 to 13% under standard culture conditions across primary and immortalized cell lines (Fig. 1*A*; Table S1) (6, 21, 34, 35, 36, 37, 38, 39). We therefore estimate that 1 × 10^−4^ to 4 × 10^−4^ pmol AcCoA per cell (∼0.5–20 times cellular AcCoA quantity) could be stored on histone tails under standard culture conditions. Thus, even partial histone deacetylation at physiological acetylation levels could potentially serve as a significant source of acetate and AcCoA. However, the fast turnover of AcCoA (half-life <5 min) and rapid flux into downstream metabolites (40) suggests that deacetylation is not likely to maintain AcCoA flux beyond a few hours. Nevertheless, the contribution of protein deacetylation to AcCoA metabolism remains to be directly tested.

### Acyl-CoA pools and Hac respond rapidly to acetate in *Acly*^*−/−*^ MEFs

Manipulating and tracing Hac is challenging because AcCoA can be generated from a wide variety of metabolic sources. To overcome this problem, we used a well-characterized cell line model in which the metabolic source of AcCoA was restricted. *Acly*^*−/−*^ MEFs are unable to efficiently use mitochondrially derived citrate to synthesize cytosolic/nuclear AcCoA and are thus primarily dependent on acetate for fatty acid synthesis (FAS) and Hac (27) (Fig. 1*B*). Although glucose is the primary source of mitochondrial metabolites in these cells, mitochondrial AcCoA does not make a substantial quantitative contribution to whole-cell levels of AcCoA, at least under standard cell culture conditions (34, 41). Critically, bulk Hac in *Acly*^*−/−*^ MEFs is sensitive to acetate supply: total Hac was lower in *Acly*^*−/−*^ MEFs than WT counterparts at physiological acetate concentration (100 μM) but increased to WT levels upon incubation with supraphysiological acetate (1 mM) (27). Furthermore, at 1 mM acetate, the vast majority of Hac (>80%) in *Acly*^*−/−*^ MEFs comes from acetate (see Table S1 for specific lysine sites) (27). We therefore reasoned that *Acly*^*−/−*^ MEFs at 1 mM acetate afforded us the unique opportunity both to manipulate the level of Hac and efficiently label the acetylation marks with just one substrate.

We first tested the dynamics of acetate flux through AcCoA and downstream products. *Acly*^*−/−*^ MEFs were incubated in 1 mM ^13^C_2_-acetate for 16 h to achieve maximal steady-state labeling of AcCoA >90%, then switched to media containing 1 mM unlabeled (UL) acetate (Fig. 1*C*). The rate of label loss for AcCoA occurred with a half-time of 1.5 min. We also observed this turnover propagated downstream into abundant acyl-CoAs (iso)butyryl-CoA (4 carbon acyl chain) and 3-hydroxy-3-methylglutaryl-CoA (HMG-CoA) (6-carbon acyl chain) with a half-time of 1.8 min and 2.8 min, respectively (Fig. 1, *C*–*E*). These turnover rates show that acetate flux through AcCoA and downstream pathways is rapid and that AcCoA is a pacemaker for these downstream pathways, in agreement with previous kinetic studies (40).

We next asked how rapidly the pool sizes of these metabolites would respond to acetate removal. Consistent with their rapid turnover, AcCoA, (iso)butyryl-CoA and HMG-CoA were depleted by more than 50% after only 30 min of acetate removal (Fig. 1, *F*–*H*). Acetate removal also slowed the turnover of AcCoA and downstream metabolites (Fig. S1, *A*–*C*). Furthermore, acetate removal led to rapid histone deacetylation: pan-acetyl lysine immunoblot of whole-cell lysate shows the most intense pan-acetyl lysine band and loss of intensity at the molecular weight corresponding to histone H3 (Fig. 1, *I* and *J*). Using quantitative histone acyl proteomics, we have previously shown that high-occupancy histone H3 sites H3K14, H3K18, and H3K23 exhibit acetylation occupancy of 44%, 11%, and 19%, respectively, in *Acly*^*−/−*^ MEFs incubated with 1 mM acetate and that this is reduced to 21%, 6%, and 9% at 0.1 mM acetate (27) (Table S1). We therefore investigated the acute deacetylation dynamics at these high-occupancy and acetate-responsive sites using specific antibodies (Fig. 1, *K* and *L*). H3K14ac and H3K23ac responded rapidly to acetate removal, with significant loss within 30 to 60 min, mirroring the AcCoA pool size, while H3K18ac reduced at a slower rate. Together, these data indicate that *Acly*^*−/−*^ MEFs provided a uniquely pliable system for manipulating and tracing AcCoA and Hac marks.

### Deacetylation does not substantially impact cellular acyl-CoA quantity

To determine the potential contribution of Hac to acyl-CoAs, we developed a pulse-chase experimental system in *Acly*^*−/−*^ MEFs in which we first maximize the amount of labeled Hac with ^13^C_2_-acetate, then wash out ^13^C label from free acyl-CoAs while maintaining the ^13^C label on Hac by inhibiting deacetylation, and finally release labeled ^13^C from histones in the absence of exogenous acetate (Fig. 2, *A* and *B*). In this experimental system, any appearance of ^13^C label in acyl-CoA pools after deacetylation likely derives from protein-bound acetate.

We first established the optimal conditions for each step. We incubated *Acly*^*−/−*^ MEFs in 1 mM ^13^C_2_-acetate, which was previously shown to achieve on an average greater than 80% labeling of Hac marks (27). To maximize labeling of Hac, we sought to inhibit deacetylation. Class I and II KDACs appeared to be the main regulators of bulk deacetylation in this system since sirtinol and nicotinamide, inhibitors of sirtuins (Class III KDAC), had no major impact on Hac when used at concentrations previously shown to inhibit sirtuin activity (42, 43, 44) (Fig. S2, *A* and *B*). Sirtuin-mediated deacetylation produces O-Acetyl-ADP-ribose, which needs to be metabolized to acetate (rather than directly generating acetate (45, 46)), further suggesting sirtuin activity is not a major contributor to protein-derived acetate. Multiple Class I and II KDAC inhibitors, however, rapidly increased Hac (Fig. S2*B*). We found that the well-characterized Class I and II KDAC inhibitor, trichostatin A (TSA) (47, 48), led to maximal acetylation after 6 h in *Acly*^*−/−*^ MEFs (Fig. S3*A*) and therefore decided to use it to inhibit deacetylation in our system.

**Figure 3:**
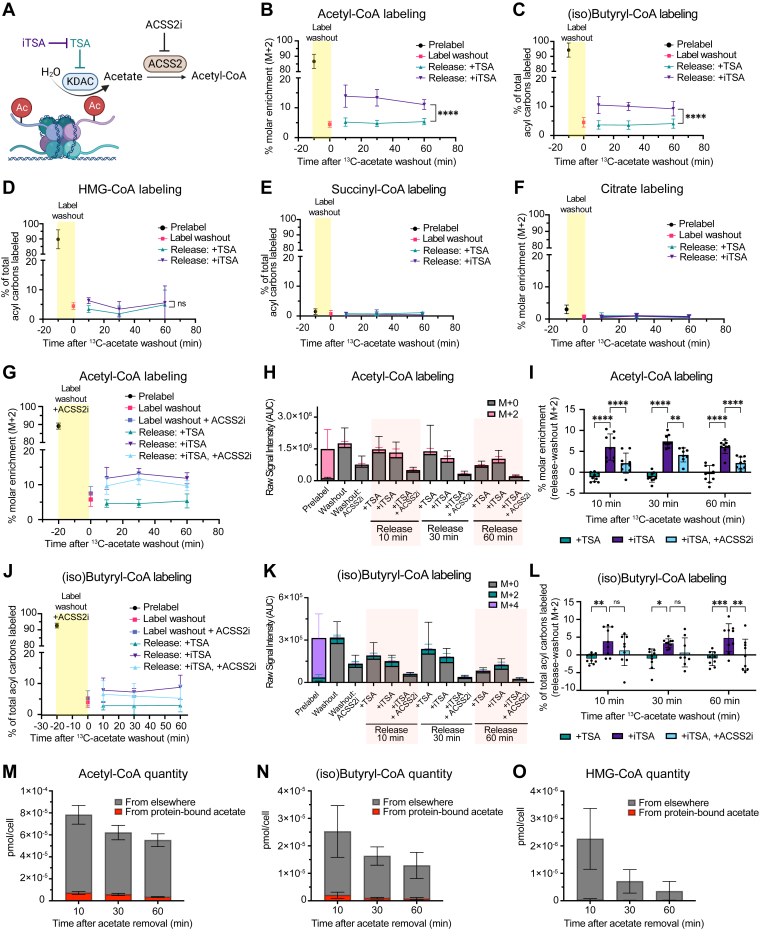
**Deacetylation contributes carbons to AcCoA and downstream pathways.***A*, schematic of histone deacetylation contributing to AcCoA. *B*–*F*, ^13^C enrichment during pulse-chase experiment. N > 9 across at least three independent experiments. *G*–*I*, AcCoA labeling during pulse-chase experiment with ACSS2i (22 μM). *G*, ^13^C enrichment (*H*) raw signal intensity (area under the curve (AUC)) for indicated isotopologues. *I*, ^13^C enrichment after washout with washout enrichment subtracted. *J*–*L*, same as (G–I) but for (iso)butyryl-CoA. N = 9 across three independent experiments. *M*–*O*, quantitation of cellular acyl-CoAs with the portion derived from protein-bound acetate indicated in *red* (calculated by multiplying percent molar enrichment by pool size at each time point). All data indicated as mean ± SD. Statistical analyses in (*B*–*D*) was by two-way ANOVA followed by Sidak’s multiple comparison test, and *I* and *L* were by two-way ANOVA followed by Dunnett’s Multiple Comparison. ns = not significant. *p* value < 0.05 (∗), *p* value < 0.01 (∗∗), *p* value < 0.001 (∗∗∗), and *p* value < 0.0001 (∗∗∗∗). A, created with BioRender.com. AcCoA, acetyl-CoA; ACSS2i, ACSS2 inhibitor.

To wash the ^13^C label out of acyl-CoAs, we switched from 1 mM ^13^C_2_-acetate to 1 mM UL acetate in the presence of TSA. ^13^C label was effectively washed out from abundant acyl-CoAs within 10 min (Fig. 3, *B*–*E*), consistent with their fast turnover (Fig. 1, *C*–*E*).

To induce deacetylation, we removed TSA and acetate from the cell culture medium. We observed that the deacetylation rate was much slower following TSA treatment (Fig. S3*B*). We reasoned this may reflect persistent TSA activity. To promote rapid deacetylation after TSA treatment, we tested the efficacy of an inhibitor of TSA (iTSA) (49). Treatment with iTSA led to deacetylation in minutes instead of hours (Fig. S3*C*). We observed no significant difference in Hac between prelabel, washout, and release steps in the presence of TSA, consistent with TSA effectively preventing deacetylation. iTSA significantly decreased Hac within 10 min of acetate removal, with the most deacetylation within 30 to 60 min (Fig. 2, *C* and *D*). Examination of specific histone H3 high-occupancy sites showed a similar response at H3K14 and H3K23, while H3K18 deacetylation was slower (Fig. S3, *D*–*E*).

These experiments led us to establish the pulse-chase system: (1) Prelabel: 18 h of incubation with 1 mM ^13^C_2_-acetate followed by 6 h of TSA treatment to maximally load protein with labeled acetate; (2) Washout: 10 min of TSA and 1 mM UL acetate to wash out the label from acyl-CoAs but maintain it on protein-bound acetate; and (3) Release: acetate removal and iTSA treatment to promote deacetylation (compared to control with acetate removal in the presence of TSA) (Fig. 2, *A* and *B*).

Surprisingly, despite the significant capacity for acetate sequestration on histones, we found no significant difference in total quantity of cellular AcCoA or other abundant short chain acyl-CoA species between deacetylation and controls (Fig. 2, *E*–*H*). Previous reports have shown that intracellular acetate can be acutely released from inside the cell into the extracellular space (17, 24). However, we found no detectable extracellular acetate after 10, 30, or 60 min of release (Fig. 2, *I* and *J*). A small amount of extracellular ^13^C_2_-acetate was detected in the label washout step likely because cells were not washed prior to switching to UL washout media. Thus, we concluded that deacetylation did not result in significant release of protein-bound acetate into the extracellular space.

### Deacetylation contributes carbons to AcCoA and downstream pathways

We next investigated the potential for protein-bound acetate to contribute to flux of acyl-CoA pools (Fig. 3*A*) by comparing ^13^C labeling of acyl-CoAs in prelabel, washout, and release steps. As expected, ^13^C enrichment was ∼90% at the prelabel step and <5% after label washout (Fig. 3, *B*–*D*). The remaining label after washout is consistent with residual labeled acetate in media during the washout step (Fig. 2*J*).

The release step revealed a significant difference in the contribution of ^13^C labeling to AcCoA between deacetylation and controls. During deacetylation (+iTSA), 15% of cellular AcCoA was labeled after 10 min of acetate removal compared to less than 5% in no release (+TSA) controls. Enhanced labeling of AcCoA persisted for at least 1-h postrelease, slowly declining to 10% enrichment (Fig. 3*B*). We observed a similar response for (iso)butyryl-CoA labeling (Fig. 3*C*). HMG-CoA labeling was marginally increased with deacetylation only at 10 min (Fig. 3*D*). Consistent with the previous observation that acetate was not a major contributor to mitochondrial AcCoA in *Acly*^*−/−*^ MEFs, there was less than 5% labeling of citrate and succinyl-CoA after the prelabel step and no labeling was detected after deacetylation (Fig. 3, *E* and *F*). Together, these data demonstrate that protein-derived acetate contributes carbons to AcCoA and proximal downstream metabolites when deacetylation is induced by acetate removal.

The conversion of acetate to AcCoA in cytosol/nucleus is largely driven by ACSS2 (24, 27). To test if appearance of label in the AcCoA pool was dependent on ACSS2 enzymatic activity, ACSS2 inhibitor (ACSS2i) was added during the label washout step (but not during the prelabel step where ACSS2 activity is required) (Fig. 3*A*). We extended the label washout from 10 to 20 min to allow sufficient time for inhibition. The washout step was still effective with ACSS2i, although there was a slight reduction in label washout efficiency (Fig. 3*G*). The amount (*i.e.*, raw signal detected) of AcCoA was dramatically decreased with ACSS2i, indicating effective inhibition (Fig. 3*H*). ACSS2i significantly reduced the appearance of label in AcCoA between washout and release steps (Fig. 3*I*). A similar pattern was observed for (iso)butyryl-CoA (Fig. 3, *J*–*L*), further suggesting that it occurs downstream of AcCoA under these conditions. These data demonstrate that ACSS2 enzymatic activity is required for deacetylation-derived acetate incorporation into AcCoA and downstream metabolites.

Finally, we calculated the quantity of deacetylation-derived acetate incorporated into AcCoA by combining isotope labeling and pool size measurements at each time point. Deacetylation contributed 7.1 × 10^−6^ pmol/cell of AcCoA 10 min after acetate removal, 9.1% of the total AcCoA pool (Fig. 3*M*). By 60 min of acetate removal, 3.4 × 10^−6^ pmol/cell of AcCoA was derived from protein-bound acetate, approximately 6% of the AcCoA pool, since both the pool size and labeling were reduced over the time course. A similar pattern was observed for (iso)butyryl-CoA: 2 × 10^−6^ pmol/cell of (iso)butyryl-CoA was derived from protein-bound acetate after 10 min of acetate removal, which decreased to 7.6 × 10^−7^ pmol/cell after 60 min (Fig. 3*N*). Finally, only about 1% of the HMG-CoA pool was derived from protein-bound acetate after 10 min of acetate removal, likely due to the inhibition of HMG-CoA synthesis upon acetate withdrawal (Fig. 3*O*). Although we cannot confidently calculate the total flux over the 60-min time course in this nonsteady state system, the maximum quantity of deacetylation-derived AcCoA detected at 10 min amounts to ∼0.3% of the maximal acetate we calculated that core histones could hold (Fig. 1*A*).

## Discussion

We directly test and quantify the potential for deacetylation to contribute to cellular AcCoA by leveraging *Acly*^*−/−*^ MEFs, which provide a uniquely pliable system for manipulating and tracing AcCoA and lysine acetylation. After inducing deacetylation of maximally acetylated histones, we detect 2-carbon labeling from deacetylation-derived acetate in 9.1% of cellular AcCoA. Consistent with direct enzymatic conversion of free acetate to AcCoA, 2-carbon labeling into AcCoA was sensitive to ACSS2 inhibition. We also observe label incorporation into 4-carbon units of (iso)butyryl-CoA, suggesting that deacetylation-derived carbons can be propagated into downstream metabolites. Surprisingly, however, this contribution represents <1% of potential contribution that we and others estimate to be on maximally acetylated histones.

Our data suggest that deacetylation is a short-lived contributor to AcCoA metabolism, on the order of minutes. We detected the highest contribution of deacetylation to AcCoA at 10 min after deacetylation, which decreased over the 60 min monitored. Deacetylation occurred predominantly between 10 and 30 min after acetate removal (Figs. 2*D* and S3*E*), indicating that deacetylation contribution to AcCoA was likely not more substantial prior to 10 min. Deacetylation also did not affect the total cellular concentration of AcCoA. Therefore, even though 50% of the AcCoA pool is sustained for at least 1 h in the absence of acetate and ACLY (Fig. 1*F*), this is likely a result of decreased turnover (Figs. 1, *C*–*E* and S1, *A*–*C*) and a contribution from alternative metabolic source(s) other than deacetylation (50, 51). Although labeled acetate could potentially be stored in fatty acids during prelabeling, the lack of labeling into citrate or succinyl-CoA (Fig. 3, *E* and *F*) implies that fatty acid oxidation does not contribute to label release in the timeframe examined in this study.

We show that HMG-CoA levels drop more than an order of magnitude 30 min after acetate removal in *Acly*^*−/−*^ MEFs, while succinyl-CoA levels are maintained (Fig. 2, *G* and *H*). Acetate removal therefore shuts down HMG-CoA–dependent anabolic pathways such as the mevalonate pathway, but mitochondrial catabolism is maintained (as reported previously (34)). We also show that protein-derived acetate contributes carbon units to (iso)butyryl-CoA in addition to AcCoA. We identify (iso)butyryl-CoA as downstream of AcCoA in our system (Figs. 1, *C* and *D* and 3, *M*–*O*). Importantly, (iso)butyryl-CoA can be an intermediate in FAS downstream of AcCoA (52). Even though the acute time points and the nutrient-depleted conditions (acetate depletion is nonlipogenic in *Acly*^*−/−*^ MEFs (27)) did not allow us to directly examine acetate carbon incorporation into lipogenesis, our data suggest that deacetylation may be used for anabolic pathways such as FAS. Future studies will illuminate the quantitative contribution of protein-derived acetate to FAS under conditions and in cell types where anabolism is activated.

Histone deacetylation may specifically enrich the nuclear compartment with protein-derived acetate (24). Although TSA inhibits both nuclear and non-nuclear KDACs, the availability of protein-derived acetate for non-nuclear uses may be limited because the acetylation stoichiometry of non-nuclear compartments is minimal and the nucleus likely operates as a distinct metabolic compartment (18, 24, 34, 53, 54). Measurements of the relative contribution of protein deacetylation to AcCoA within specific subcellular compartments would be required to investigate the potential for compartment-specific functions of protein/histone deacetylation.

The rapid response of Hac to acetate in *Acly*^*−/−*^ MEFs emphasizes the dynamic nature of posttranslational lysine acetylation (22, 23). Hac promotes gene expression through at least two mechanisms: (1) chromatin decompaction by neutralizing lysine-positive charge on histones (55) and (2) protein interactions recruiting transcriptional machinery (56). Our data show differential sensitivity of specific histone lysine sites to AcCoA, consistent with previous studies in mammalian cells (10, 57). Furthermore, genome-wide studies of Hac dynamics in yeast revealed that H3K14 and H3K23 functioned as acetate reservoirs, while other lysines were site-specifically regulated to mediate rapid changes in transcription (26). Although we observe >50% loss of acetylation at histone H3K14 and H3K23, acetylation at H3K18 and of other proteins (assessed by pan Ac-K) was mostly retained at the time points examined in this study, even in the absence of acetate (Figs 1, *K* and *L* and S3, *D*–*E*). Thus, our data may be consistent with a model in which the acetate sequestered on histones is preferentially mobilized to maintain other acetylation events rather than being used in metabolism. Whether acetate-responsive Hac in *Acly*^*−/−*^ MEFs preferentially affects specific genome regions and gene expression is an interesting question for future investigation.

Limitations of this study include that our experimental system is not physiological, and the measurement of total Hac may not accurately reflect chromatin occupancy due to the possibility of site-specific bias of antibodies and other quantitative limitations of immunoblot. Overall, the functional consequence of the relatively minor contribution (9.1%) of deacetylation to AcCoA is unclear. Future studies testing the effect on viability, differentiation, or other functional outputs in cell types exposed to variable nutrient supply (such as endothelial or cancer cells) would be of interest. Coupling measurements of compartment-specific contribution of deacetylation with chromatin-wide acetylation patterns would give added insight into the origin and function of deacetylation-derived acetate and begin to shed light on why it has limited potential for maintaining cellular AcCoA metabolism.

## Experimental procedures

Full experimental procedures can be found in the supporting information and Table S3: Acyl-CoA masses used for LC/MS acyl-CoA quantification.

## Data availability

All data are contained in the article.

## Supporting information

This article contains supporting information (58, 59, 60, 61).

## Conflict of interest

The authors declare that they have no conflicts of interest with the contents of this article.
