# Supplementary material for: Dynamic protein deacetylation is a limited carbon source for acetyl-CoA–dependent metabolism
Source: J Biol Chem. 2023 May 2;299(6):104772. doi: 10.1016/j.jbc.2023.104772 (PMC10244699; doi:10.1016/j.jbc.2023.104772)
Supplement: Supporting information [file mmc4.docx]

**Supporting Information**

**Table of Contents**

[Fig S1 2](#_Toc132631704)

[Fig S2 3](#_Toc132631705)

[Fig S3 4](#_Toc132631706)

[Supplemental Table Descriptions 6](#_Toc132631707)

[Experimental Procedures 7](#_Toc132631708)

[Abbreviations 12](#_Toc132631709)

Fig S1**. Acetate removal slows acyl-CoA turnover in *Acly^-/-^*MEFs. (A)** Percent molar enrichment (M+2) of AcCoA after 1mM acetate removal. **(B)** Percent total acyl carbons labeled of (iso)butyryl-CoA after 1mM acetate removal. **(C)** Same as in **(B)** but for HMG-CoA. N>6 across at least 2 independent experiments**.**

Fig S2**. Class III KDAC inhibition has minimal effect on global Hac.(A)**IB of pan-Kac in acid-extracted histones after pretreatment with acetate (1 mM) for 16hfollowed by acetate removal in the presence of Sirtinol (50μM), Apicidin (1μM), TSA (300nM), Panobinostat (400nM) or sodium butyrate (2mM). **(B)**IB of pan-Kacfor acid extracted histones from cell pretreated with acetate (1 mM) for 16hfollowed by acetate removal in the presence of nicotinamide (5mM), sodium butyrate (2mM), Panobinostat (400nM), Apicidin (1μM) or TSA (300nM) at indicated timepoints. Controls without media change are included for 0.1mM and 1mM acetate pretreatment.

**
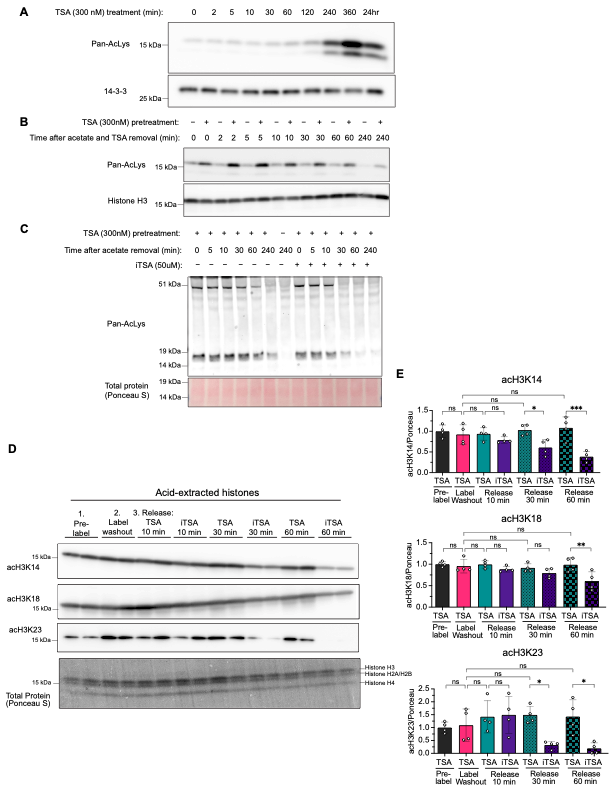
**

Fig S3**.iTSA speeds up reversal of TSA. (A)** IB of pan-Kac after indicated timepoint of treatment with TSA (300nM). **(B)** IB of pan-Kac upon TSA and acetate removal after pretreatment with TSA (300nM) for 10 min in the presence of 1mM acetate. **(C)**IB of pan-Kac upon TSA removal with or without iTSA (50μM) after pretreatment with TSA (300nM) in the presence of 1mM acetate for 6h.**D)** Representative IB of indicated histone lysine sites of acid-extracted histones. **(E)** Quantification of each lysine site in (D) normalized to total protein (Ponceau S). N=4 across 2 independent experiments. Statistical analysis in **E** was by one-way ANOVA followed by Tukey’s Multiple Comparison test withrelevant comparisons indicated. ns = not significant. P-value <0.05 (*), p-value <0.01 (**), p-value <0.001 (***), P-value <0.0001 (****).

Supplemental Table Descriptions**:**

**Table S1**: Maximal and physiological acetate amount on N-terminal histone tails and acetylation occupancy of *Acly^-/-^*MEFs.

**Table S2:** Distribution of acetylation across subcellular compartments.

**Table S3**: Acyl-CoA masses used for LC/MS acyl-CoA quantification.

# Experimental Procedures

**Cell culture**

*Acly^-/-^* MEFs were generated in the Wellen lab as reported previously(27).*Acly^-/-^* MEFs were maintained at <80% confluence (passaged every 3-4 days) in high glucose DMEM (Gibco #11965-084) with 10% fetal bovine serum (FBS, Hyclone cat# SH30071.03). *Acly^-/-^* MEFs were negative for mycoplasma, tested through the University of Pennsylvania Cell Center Mycoplasma Testing Service.

**Pulse-chase experimental system**

*Acly^-/-^* MEFs were seeded equally in 6cm dishes and grown to 80% confluence. Cells were washed with phosphate buffered saline (PBS, Corning #21-040-CM) and protein acetylation was prelabeled by incubation in DMEM(Gibco #11965-084) + 10% dialyzed FBS (dFBS, GeminiBio #100-108) + 1mM ^13^C_2_ - acetate (Cambridge Isotope #CLM-440-0) for 18 hours (27). Prelabeling media was removed by aspiration and maximal acetylation induced by incubation in DMEM + 10% dFBS + 1mM ^13^C_2_ – acetate + 300 nM TSA (Selleck Chemicals #S1054) for 6 hours. Media was removed by aspiration and replaced with UL media DMEM + 10% dFBS + UL acetate 1mM(Sigma #S7547) + 300 nM TSA for 10 min. Finally, cells were washed with PBS and media replaced with DMEM + 10% dFBS + 300nM TSA (control) or DMEM + 10% dFBS + 50μM iTSA1 (Cayman Chemical #18681) (acetylation release). Metabolism was quenchedby direct extraction (described below). UL control dishes were treated as described above but with UL acetate instead of ^13^C-acetate.Fractional enrichment was calculated by normalization to UL controls within each experiment.

When ACSS2 inhibitor (Selleck #S8588, CAS No. 508186-14-9) was added, the experiment above was altered in three ways: 1) the label washout period was increased to 20 minutes; 2) an additional label washout group was included that consisted of DMEM + 10% dFBS + 1mM ^12^C – acetate + 300 nM TSA + 22μM ACSS2i; and 3) an additional release group was included consisting of DMEM + 10% dFBS + 50 μM iTSA1 + 22μM ACSS2i.

For acyl-CoA pool size quantification, at least one dish per experimental condition was used for quantifying cell number (Corning #6749).

**Metabolite extraction from cells**

Direct extraction of whole cell metabolites was carried out as described previously(40). For short-chain acyl-CoA detection, media was poured into a waste container, dishes were placed on ice and residual media was aspirated. 1ml of ice-cold 10% (w/v in water) trichloroacetic acid(TCA) (Sigma #T6399) was added immediately to each dish. For acyl-CoA pool size quantification, samples were spiked with 0.1 mL of ^13^C_3_^15^N_1_-acyl-CoA internal standard prepared from yeast as previously described(58) and calibration curves were prepared from commercially available acyl-CoA standards (Sigma Aldrich). Cells were scraped, transferred into 1.5 ml tubes on ice, and frozen at -80°C until further processing.

For citrate detection, media was quickly aspirated, cells were washed with 2ml ice-cold PBS and then 1ml of 80:20 HPLC grade methanol:water (Optima) cooled at -80°C was added. Cells were scraped while on dry ice, transferred into 1.5 ml tubes and frozen at -80°C until further processing.

**Immunoblotting**

Immunoblots were conducted on protein precipitated by direct 10% TCA extraction. Protein pellets were spun at 13,000xg, 10 minutes at 4°C, washed with 1mL acetone, spun down again, then acetone was decanted and pellet was allowed to dry. Pellets were resuspended in 80μl 2% SDS in 10mM Tric-HCL pH 7.5 and vortexed/incubated at 37°C until dissolved. Supernatants were taken after centrifugation at 10,000xg, 5 minutes at room temperature to eliminate insoluble matter. Each sample’s protein content wasadjusted to equal concentration with Pierce BCA Kit (Thermo Scientific #23225)and then boiled at 95°C, 10 minutes in Laemmli buffer (Bio-Rad #161-0747).

Samples (20-30μg of protein) were run on a 4-20% gradient tris-polyacrylamide gel (Bio-Rad #3450034),100 volts for 1.25 hours. Protein was transferred onto PVDF membranes (Millipore #IPVH00010), which were then incubated with Ponceau (0.1% w/v Ponceau S (Sigma #P3504) in 5% glacial acetic acid) for 10 minutes and imaged (GE ImageQuant LAS 4000) on digitization (epi-illumination) mode. Membranes were blocked in 5% non-fat dry milk in tris-buffered saline, 0.1%-tween (TBST) for 30 minutes and incubated in primary antibody overnight. After 3x10mins washes in TBST, membranes were incubated for 60 minutes in rabbit horseradish peroxidase-conjugated secondary (1:10,000 Cell Signaling Technology #7074) and washed again with TBST 3x10 minutes. Enhanced chemiluminescent HRP substrate (Thermo #34095) was used to determine protein signal. Pan-Kac primary antibody (ICP #0380) was used for Fig.S3C; pan-Kac (CST #9441) was used for all other immunoblots.

In Fig. 1K-L and Fig. S3D-E, histones were acid-extracted as described previously (8, 27). anti-H3K14 (CST #7627), anti-H3K18 (CST #9675), anti H3K23 (CST #14932) were used.

Immunoblots were quantified with Fiji (59). Mean gray value for each sample lane was determined, and mean gray value for an empty lane was used as background subtraction. Each background subtracted value was divided by each sample’s specific ponceau signal (mean gray value) to account for loading variability.

**Acyl-CoA analysis by liquid-chromatography mass spectrometry**

Cell samples in 10% (w/v) TCA in water were sonicated for 12 × 0.5 s pulses, and protein was pelleted by centrifugation at 13,000 ×*g* from 10 min at 4 °C. The supernatant was purified by solid-phase extraction using Oasis HLB 1cc (30 mg) SPE columns (Waters). Columns were washed with 1 mL methanol, equilibrated with 1 mL water, loaded with supernatant, desalted with 1 mL water, and eluted with 1 mL methanol containing 25 mM ammonium acetate. The purified extracts were evaporated to dryness under nitrogen then resuspended in 55 μl 5% (w/v) 5-sulfosalicylic acid (SSA) in water.

5–10 μl of purified samples in 5% SSA were analyzed by injection of an Ultimate 3000 Quaternary Ultra High Pressure Liquid Chromatograph coupled to a QExactive Plus (Thermo Scientific) mass spectrometer in positive ESI mode using the settings described previously(60). Quantification of acyl-CoAs was via their [M+H]+ ions, and isotope tracing via their MS2 product ion from the predominant [M+H-507]+ neutral loss fragment with the targeted masses used for isotopologue analysis are indicated in **Table S3**. Data were integrated using Tracefinder v4.1 (Thermo Scientific) software. Isotopic enrichment in tracing experiments was calculated by normalization to UL control samples using the FluxFix online application(61).

**Acetate quantitation by gas-chromatography mass spectrometry**

For extracellular acetate measurement, 1mL of media was centrifuged at 2500xg, 5 minutes and then frozen at -80°C until further processing. Acetate measurement achieved by as previously described (4) with minor adjustments.200μL of thawed media was added to a 2mL eppendorf tube followed by 40μL of 1mM ^13^C_2_-D_3_ sodium acetate (Cambridge Isotope #CDLM-3457) internal standard, 50μL of 1-propanol and 50μL of pyridine were added, and samples were incubated on ice for 5 minutes. 100μL of sodium hydroxide (1M) was added, immediately followed by 30μL methyl chloroformate. Samples were vigorously vortexed for 20 seconds, pausing to vent halfway through. Finally, 300μL of methyl tert-butyl ether was added, samples were vortexed for 20 seconds and centrifuged at 10,000 x g for 5 minutes at 4°C. 100μL of the upper layer was transferred to gas-chromatography vials for analysis.

Agilent 7890B gas chromatograph (GC) coupled with an Agilent 5977B mass selective detector (MSD) was used for analysis. The GC column was a 30 m x 250 μm x 0.25 μm HP-5ms Ultra Inert column (Agilent #19091S-433UI). 2μL of sample was injected at 15mL/min split flow (15:1 split ratio), with inlet temperature at 280°C. The oven was held at 45°C for 0.8 minutes, ramped from 45°C to 60°C at 25°C/min and held for 0 minutes, then ramped from 60°C to 190°C at 50°C /min and held for 0 min. The MSD was operated in SIM mode for m/z 61 (^12^C-acetate), 63 (^13^C-acetate) and 66 (^13^C_2_; D_3_-acetate) with a dwell time of 50ms for each ion. Agilent MassHunter Quantitative Analysis software (B.07.00) was used for peak area integration. Peak area of m/z 61 and 63 for each sample was divided by m/z 66 to normalize to the internal standard. In each experiment, background ^12^C-acetate was subtracted out of all samples by using media blank, i.e. DMEM + 10% dFBS.

# Abbreviations

Histone acetylation (Hac)

Acetyl-coenzyme-A (AcCoA)

Lysine deacetylase (KDAC)

Immunoblot (IB)

Acetyl-lysine (Kac)

Mouse Embryonic Fibroblasts (MEF)

Trichloroacetic Acid (TCA)

acyl-CoA synthetase short chain family member 2 (ACSS2)

ATP citrate lyase (ACLY)

Unlabeled (UL)

ACSS2 inhibitor (ACSS2i)

Trichostatin A (TSA)

inhibitor of TSA (iTSA)

Fetal Bovine Serum (FBS)

Dialyzed Fetal Bovine Serum (dFBS)
